# Supplementary material for: Association between Ready-to-Eat Cereal Consumption and Nutrient Intake, Nutritional Adequacy, and Diet Quality among Infants, Toddlers, and Children in the National Health and Nutrition Examination Survey 2015–2016
Source: Nutrients. 2019 Aug 23;11(9):1989. doi: 10.3390/nu11091989 (PMC6769511; doi:10.3390/nu11091989)
Supplement: Supplementary file 1 [file nutrients-11-01989-s001.pdf]

Supplementary Materials

Table S1. Day-1 versus day-2 24-h dietary recall status for children 0.5 to 17 years, NHANES 2015-2016.

		**Day 1 24-h dietary recall status**				
		Reliable and met the minimum criteria	Not reliable or not met the minimum criteria	Reported consuming breast-milk	Not done	Total
**Day 2 24-h recall status**	Reliable and met the minimum criteria	2381 (65.8%)	0 (0%)	5 (0.1%)	0 (0%)	2386 (65.9%)
	Not reliable or not met the minimum criteria	20 (0.6%)	4 (0.1%)	0 (0%)	63 (1.7%)	67 (1.9%)
	Reported consuming breast-milk	0 (0%)	0 (0%)	74 (2.0%)	0 (0%)	74 (2.0%)
	Not done	568 (15.7%)	63 (1.7%)	9 (0.2%)	496 (13.7%)	1136 (31.4%)
	Total	2969 (82.0%)	67 (1.9%)	89 (2.5%)	496 (13.7%)	3621 (100.0%)

Table S2. Sensitivity analysis using two days of dietary intake data: Nutrient intakes for children 0.5 to 17 years who were ready-to-eat (RTE) cereal non-eaters, one-day RTE cereal eaters and two-day RTE cereal eaters in the National Health and Nutrition Examination Survey (NHANES) 2015-2016.[1]

**NHANES cycle**	**All Children (≥0.5 to ≤17 years)**			
	**Non-RTE cereal eaters**	**One-day RTE cereal eater**	**Two-day RTE cereal eater**	**p value[2]**
n (%)	1136 (47.5%)	814 (32.6%)	431 (20.0%)	
Energy, kcal[3]	1722	1771	1792	0.36
Total Carbohydrate, g	216[a]	224[b]	233[c]	**<0.0001**
Total Sugar, g	96[a]	100[b]	105[b]	**0.0001**

Added sugar, tsp. eq.	12	13	14	0.02
Fiber, g	13[a]	14[b]	15[b]	**0.0003**
Total Fat, g	68[a]	65[b]	62[c]	**<0.0001**
Saturated Fat, g	24[a]	23[b]	23[b]	0.006
Protein, g	63	63	62	0.67
Calcium, mg	890[a]	964[b]	1049[c]	**0.0002**
Iron, mg	10[a]	14[b]	18[c]	**<0.0001**
Magnesium, mg	212[a]	221[b]	231[c]	**0.0003**
Potassium, mg	1995	2084	2102	0.01
Sodium, mg	2770	2713	2628	0.02
Zinc, mg	7.9[a]	9.8[b]	12.0[c]	**<0.0001**
Vitamin A, µg RAE	490[a]	612[b]	764[c]	**<0.0001**
Thiamin, mg	1.2[a]	1.5[b]	1.8[c]	**<0.0001**
Riboflavin, mg	1.6[a]	1.9[b]	2.2[c]	**<0.0001**
Niacin, mg	17[a]	21[b]	23[c]	**<0.0001**
Vitamin B_6, mg	1.3[a]	1.7[b]	2.1[c]	**<0.0001**
Folate, µg DFE	346[a]	517[b]	697[c]	**<0.0001**
Vitamin B_{12}, µg	3.4[a]	4.8[b]	6.2[c]	**<0.0001**
Vitamin C, mg	73	77	79	0.31
Vitamin D, µg	4.7[a]	6.2[b]	7.4[c]	**<0.0001**
Vitamin E, mg	6.8	6.7	6.7	0.89

DFE, dietary folate equivalents; NHANES, National Health and Nutrition Examination Survey; RAE, retinol activity equivalents; tsp eq., teaspoon equivalents; [1]Data are based on the National Health and Nutrition Examination Survey 2015-2016 data. [2]RTE cereal non-eaters were defined as those that did not report consuming cereal on day 1 or day 2 24-h dietary recalls; one day RTE cereal eaters were those that reported consuming any quantity of RTE cereal on one 24-h recall but not the other; and two day RTE cereal eaters were those that reported consuming reported consuming any quantity of RTE cereal on both 24-hr recalls. [2]Data were adjusted for age, gender, poverty to income ratio (PIR), ethnicity and energy intake. We applied the Bonferroni corrected p-value from our main analysis (Table 2) of 0.0007 to set our statistical level of significance. P values that met the level of statistical significance are in bold text. [3]Energy intake was only adjusted for age, gender, PIR and ethnicity.

Table S3. Number and percentage of participants by age from 2003 to 2016; results from 2003-2004 to 2015-2016 National Health and Nutrition Examination Surveys (NHANES)[1].

NHANES cycle	Infants and Toddlers (0.5 to ≤2 years)		Children (≥2 to ≤12 years)		Adolescents (≥13 to ≤17 years)		All Children (≥0.5 to ≤17 years)	
	RTE cereal eaters[2]	RTE cereal non-eaters	RTE cereal eaters	RTE cereal non-eaters	RTE cereal eaters	RTE cereal non-eaters	RTE cereal eaters	RTE cereal non-eaters
2003-2004 n (%)	166 (42%)	341 (58%)	866 (46%)	1029 (54%)	343 (29%)	995 (71%)	1375 (41%)	2365 (59%)
2005-2006 n (%)	178 (42%)	322 (58%)	986 (46%)	1182 (54%)	371 (31%)	908 (69%)	1535 (41%)	2412 (59%)
2007-2008 n (%)	149 (32%)	323 (68%)	925 (43%)	1154 (57%)	210 (30%)	530 (70%)	1284 (38%)	2007 (62%)
2009-2010 n (%)	151 (36%)	313 (64%)	974 (43%)	1164 (57%)	238 (28%)	586 (72%)	1363 (38%)	2063 (62%)
2011-2012 n (%)	100 (36%)	236 (64%)	908 (43%)	1213 (57%)	196 (24%)	538 (76%)	1204 (37%)	1987 (63%)
2013-2014 n (%)	94 (33%)	268 (67%)	747 (41%)	1147 (59%)	212 (28%)	600 (72%)	1053 (37%)	2015 (63%)
2015-2016 n (%)	88 (33%)	236 (67%)	688 (37%)	1169 (63%)	242 (34%)	546 (66%)	1018 (36%)	1951 (64%)
All cycles combined 2003-2016 n (%)	926 (36%)	2039 (64%)	6094 (43%)	8058 (57%)	1812 (29%)	4703 (71%)	8832 (38%)	14800 (62%)

RTE, ready-to-eat. [1] Data are based on the NHANES data from 2003-2004 to 2015-2016. [2]RTE cereal eaters were defined as those that reported consuming any quantity of RTE cereal on their day 1 24-hr recall.

Table S4. Nutrient intakes, unadjusted, comparing ready-to-eat (RTE) cereal eaters and non-eaters among children in the National Health and Nutrition Examination Survey (NHANES) 2015-2016.[1]

NHANES cycle	Infants and Toddlers (0.5 to ≤2 years)			Children (≥2 to ≤12 years)			Adolescents (≥13 to ≤17 years)			All Children (≥0.5 to ≤17 years)		
	RTE cereal eaters[2]	RTE cereal non-eaters	p value	RTE cereal eaters[2]	RTE cereal non-eaters	p value	RTE cereal eaters[2]	RTE cereal non-eaters	p value	RTE cereal eaters[2]	RTE cereal non-eaters	p value
Energy, kcal	1170	1107	0.37	1711	1765	0.30	2079	2066	0.90	1788	1817	0.57
Total Carbohydrate, g	152	141	0.20	234	223	0.13	282	254	0.03	244	228	0.02
Total Sugar, g	83	82	0.92	109	99	0.04	128	113	0.06	113	102	0.02
Added sugar, tsp. eq.	5.5	4.4	0.07	15	14	0.41	19	18	0.43	16	15	0.30
Fiber, g	8.3	7.5	0.15	14	13	0.20	17	15	0.002	14	13	0.007
Total Fat, g	44	44	0.98	62	71	0.0007	77	84	0.16	65	73	0.003

Saturated Fat, g	18	18	0.92	22	25	0.003	28	29	0.72	24	26	0.02
Protein, g	44	39	0.19	61	63	0.28	72	78	0.25	63	66	0.24
Calcium, mg	971	879	0.20	1036	916	0.003	1184	935	0.0004	1076	920	<0.0001
Iron, mg	11	11	0.92	17	11	<0.0001	22	12	<0.0001	18	11	<0.0001
Magnesium, mg	164	145	0.10	224	214	0.21	275	248	0.09	235	220	0.07
Potassium, mg	1719	1596	0.32	2085	1958	0.06	2352	2199	0.27	2142	2011	0.08
Sodium, mg	1531	1309	0.12	2587	2861	0.0007	3232	3475	0.27	2715	2954	0.02
Zinc, mg	4.8	6.6	0.05	11.2	8.0	<0.0001	14.0	10.1	0.001	11.8	8.6	<0.0001
Vitamin A, μg RAE	637	604	0.46	739	503	<0.0001	833	490	<0.0001	761	505	<0.0001
Thiamin, mg	1.1	0.9	0.006	1.7	1.3	<0.0001	2.2	1.5	<0.0001	1.8	1.3	<0.0001
Riboflavin, mg	1.8	1.4	0.004	2.2	1.6	<0.0001	2.6	1.8	<0.0001	2.3	1.7	<0.0001
Niacin, mg	13	10	0.06	23	17	<0.0001	29	23	0.003	24	19	<0.0001
Vitamin B_6, mg	1.3	0.9	0.003	2.0	1.3	<0.0001	2.5	1.6	<0.0001	2.1	1.4	<0.0001
Folate, μg DFE	405	234	0.0003	659	359	<0.0001	818	424	<0.0001	691	372	<0.0001
Vitamin B_{12}, μg	4.5	3.1	0.002	5.8	3.3	<0.0001	7.4	4.2	<0.0001	6.2	3.5	<0.0001
Vitamin C, mg	68	77	0.33	76	63	0.003	76	64	0.16	75	64	0.01
Vitamin D, μg	8.6	8.1	0.45	7.3	4.5	<0.0001	7.9	3.8	<0.0001	7.5	4.5	<0.0001
Vitamin E, mg	4.1	5.9	0.002	6.6	6.8	0.62	8.2	8.1	0.87	6.9	7.2	0.56

DFE, dietary folate equivalents; NHANES, National Health and Nutrition Examination Survey; RAE, retinol activity equivalents; tsp eq., teaspoon equivalents; [1] Data are based on the National Health and Nutrition Examination Survey 2015-2016 data. [2]RTE cereal eaters were defined as those that reported consuming any quantity of RTE cereal on their day 1 24-hr recall.

Supplemental Methods: Percentage of the Population Below the Estimated Average Requirements and Above the Upper Level

Using the Dietary Reference Intake (DRI), we calculated the percentages below the Estimated Average Requirement (EAR) for thiamin, riboflavin, niacin, vitamin B6, folate, vitamin B12, vitamin A, vitamin C, vitamin D, iron, zinc and calcium, as well as the percentage above the Upper Level (UL) for dietary fiber based on the National Cancer Institute (NCI) Usual Intake Method using both day 1 and day 2 24-h recall dietary data [1,2]. Covariates in the NCI usual intake model included they weekday of dietary intake collection (i.e. weekday or weekend) and the interview sequence of the 24-hr recall. The percent below EAR or above UL were calculated according to the DRI age/sex groups and then combined into the age groups used in the current analysis. We did not calculate the percent below the EAR or above the UL for infants and toddlers 0.5 to 2 years because there are no EARs set for the age group 6 months to 12 months.

Table S5. Percentage of children 2 to 17 years below the Estimated Average Requirement (EAR) for select nutrients by ready-to-eat cereal consumption status using data from the National Health and Nutrition Examination Survey (NHANES) 2015-2016.[1]

**Nutrient**	**Children (≥2 to ≤12 years)**		**Adolescents (≥13 to ≤17 years)**		**All Children[2] (≥2 to ≤17 years)**	
	RTE cereal eaters[3] Below EAR, %	RTE cereal non-eaters Below EAR, %	RTE cereal eaters[3] Below EAR, %	RTE cereal non-eaters Below EAR, %	RTE cereal eaters[3] Below EAR, %	RTE cereal non-eaters Below EAR, %
Vitamin A	0.4	18.1	10.3	68.8	3.4	35.4
Thiamin	0	0	0.6	9.2	0.2	3.1
Riboflavin	0	0.3	0.2	6.7	0.1	2.5
Niacin	0	0.2	0.2	1.6	0.1	0.7
Vitamin B_6	0	0.1	0.5	8.1	0.1	2.8
Folate	0	0.1	0	24.8	0	8.6
Vitamin B_{12}	0	0.5	0.1	6.6	0	2.6
Vitamin C	4.6	11.9	33.7	50.2	13.1	25.1
Vitamin D	88.5	97.7	77.0	97.7	84.6	97.8
Calcium	23.1	44.1	42.8	72.5	29.4	53.7
Iron	0	0.1	0	10.9	0	3.8
Zinc	0.8	3.5	5.8	23.8	2.2	11.1

EAR, estimated average requirement; RTEC, ready-to-eat cereal. [1] Data are from the National Health and Nutrition Examination Survey 2015-2016. Children 0.5 to 17 years with complete Day 1 24-hr dietary recalls were included in the analysis. [2] We did not calculate the percent below the EAR for infants and toddlers 0.5 to 2 years because there are no EARs set for the age group 6 months to 12 months. [3] RTEC eaters were defined as those children that reported consuming any quantity of RTEC on their day 1 24-hr dietary recall.

Table S6. Percent contribution to daily nutrient intakes of ready-to-eat (RTE) cereal and milk (when co-consumed) among children 0.5 to 17 years who reported consuming ready-to-eat (RTE) cereal in the National Health and Nutrition Examination Survey (NHANES) 2015-2016.[1]

Nutrient	Infants and Toddlers (0.5 to ≤2 years)	Children (≥2 to ≤12 years)	Adolescents (≥13 to ≤17 years)	All Children (≥0.5 to ≤17 years)
Energy	7.3	13.6	14.3	13.6
Folate	49.3	55.6	56.3	55.6
Vitamin B_{12}	26.8	53.1	54.7	52.6
Iron	40.4	52.3	52.2	51.9
Whole grains	47.7	46.1	51.4	48.0
Vitamin D	12.5	47.8	53.3	47.1
Vitamin B_6	32.0	45.9	49.0	46.5
Vitamin A	21.2	44.7	49.8	45.2
Riboflavin	15.5	39.6	43.3	39.7
Thiamin	25.4	39.4	41.1	39.5
Zinc	28.5	39.2	41.1	39.5
Niacin	28.1	35.7	36.7	35.8
Calcium	9.5	25.7	27.2	25.3
Added sugar	16.2	17.7	19.0	18.2
Total sugar	7.1	17.8	19.8	18.0
Carbohydrate	10.4	17.4	18.8	17.6
Fiber	14.8	16.5	19.7	17.6
Magnesium	10.0	17.5	18.5	17.5
Potassium	6.3	15.3	16.4	15.3
Vitamin E	3.0	13.3	15.6	13.8
Vitamin C	5.5	12.4	17.3	13.5
Protein	6.0	13.0	13.2	12.8
Sodium	6.9	10.7	10.7	10.6
Saturated fat	4.0	10.9	10.1	10.3
Total fat	3.9	8.1	8.2	7.9

[1] Data are from the National Health and Nutrition Examination Survey 2015-2016. Children 0.5 to 17 years with complete Day 1 24-hr dietary recalls who reporting consuming any quantity of RTE cereal on the day 1 24-hr recall were included in the analysis.

Table S7. Intake of protein food group subcategories for children 0.5 to 17 years, by RTE cereal eating status from the Food Patterns Equivalents Database 2015-2016[1]

	**Infants and Toddlers (0.5 to 2 years)**			**Children (>2 to 12 years)**			**Adolescents (13 to 17 years)**			**All Children (0.5 to 17 years)**		
	RTEC eaters[2]	RTEC non-eaters	p value[3]	RTEC eaters[2]	RTEC non-eaters	p value[3]	RTEC eaters[2]	RTEC non-eaters	p value[3]	RTEC eaters[2]	RTEC non-eaters	p value[3]
Total protein foods including legumes (oz eq.)	1.31 ± 0.14	1.77 ± 0.10	0.02	3.68 ± 0.15	4.30 ± 0.12	0.009	4.07 ± 0.24	5.69 ± 0.25	**<0.0001**	3.56 ± 0.11	4.41 ± 0.10	**0.0001**
Meat, poultry, and seafood (oz eq.)	1.10 ± 0.16	1.26 ± 0.10	0.42	2.71 ± 0.12	3.09 ± 0.09	0.03	3.11 ± 0.22	4.36 ± 0.26	**0.0002**	2.65 ± 0.08	3.23 ± 0.09	**0.0005**
Eggs (oz eq.)	0.16 ± 0.04	0.22 ± 0.04	0.27	0.30 ± 0.08	0.47 ± 0.08	**0.0005**	0.28 ± 0.12	0.43 ± 0.08	0.05	0.28 ± 0.07	0.44 ± 0.06	**0.0009**
Nuts and seeds (oz eq.)	0.02 ± 0.09	0.09 ± 0.04	0.55	0.38 ± 0.12	0.38 ± 0.11	0.98	0.36 ± 0.20	0.43 ± 0.17	0.50	0.34 ± 0.08	0.37 ± 0.08	0.69
Soybean products (oz eq.)	0.01 ± 0.01	0.00 ± 0.01	0.27	0.04 ± 0.01	0.03 ± 0.02	0.89	0.01 ± 0.12	0.07 ± 0.10	0.05	0.03 ± 0.05	0.04 ± 0.05	0.25
Legumes (oz eq.)	0.03 ± 0.08	0.19 ± 0.05	0.08	0.25 ± 0.05	0.33 ± 0.04	0.08	0.31 ± 0.16	0.40 ± 0.13	0.29	0.25 ± 0.08	0.33 ± 0.06	0.07

[1] Data are from the Food Pattern Equivalent Database (FPED) 2015-2016. Children 0.5 to 17 years with complete Day 1 24-hr dietary recalls were included in the analysis. Results are adjusted for demographic characteristics and energy intake. [2]RTE cereal eaters were defined as those that reported consuming any quantity of RTE cereal on their day 1 24-hr recall. [3] *P* values were calculated using multivariable linear regression and $p \leq 0.002$ was considered statistically significant (based on main analysis found in Table 3); *p* values that met the level of statistical significance are in bold.

Supplementary References

1. Kipnis, V.; Midthune, D.; Freedman, L.; Bingham, S.; Day, N.E.; Riboli, E.; Ferrari, P.; Carroll, R.J. Bias in dietary-report instruments and its implications for nutritional epidemiology. Public Health Nutr 2002, 5, 915-923, doi:10.1079/PHN2002383.

2. Dodd, K.W.; Guenther, P.M.; Freedman, L.S.; Subar, A.F.; Kipnis, V.; Midthune, D.; Tooze, J.A.; Krebs-Smith, S.M. Statistical methods for estimating usual intake of nutrients and foods: a review of the theory. J Am Diet Assoc 2006, 106, 1640-1650, doi:10.1016/j.jada.2006.07.011.
